# Common and Rare Variant Analysis in Early-Onset Bipolar Disorder Vulnerability

**DOI:** 10.1371/journal.pone.0104326

**Published:** 2014-08-11

**Authors:** Stéphane Jamain, Sven Cichon, Bruno Etain, Thomas W. Mühleisen, Alexander Georgi, Nora Zidane, Lucie Chevallier, Jasmine Deshommes, Aude Nicolas, Annabelle Henrion, Franziska Degenhardt, Manuel Mattheisen, Lutz Priebe, Flavie Mathieu, Jean-Pierre Kahn, Chantal Henry, Anne Boland, Diana Zelenika, Ivo Gut, Simon Heath, Mark Lathrop, Wolfgang Maier, Margot Albus, Marcella Rietschel, Thomas G. Schulze, Francis J. McMahon, John R. Kelsoe, Marian Hamshere, Nicholas Craddock, Markus M. Nöthen, Frank Bellivier, Marion Leboyer

**Affiliations:** 1 Institut National de la Santé et de la Recherche Médicale U955, Psychiatrie Génétique, Créteil, France; 2 Université Paris-Est, Faculté de Médecine, Créteil, France; 3 Fondation FondaMental, Créteil, France; 4 Institute of Neuroscience and Medicine (INM-1), Research Center Juelich, Juelich, Germany; 5 Institute of Human Genetics, University of Bonn, Bonn, Germany; 6 Department of Genomics, Life and Brain Center, University of Bonn, Bonn, Germany; 7 Division of Medical Genetics, University Hospital and Department of Biomedicine, University of Basel, Basel, Switzerland; 8 Assistance Publique - Hôpitaux de Paris, Hôpital Henri Mondor-Albert Chenevier, Pôle de Psychiatry, Créteil, France; 9 Department of Genetic Epidemiology in Psychiatry, Central Institute of Mental Health, Mannheim, Germany; 10 Department of Psychiatry, University of Bonn, Bonn, Germany; 11 Assistance Publique - Hôpitaux de Paris, Hôpital Henri Mondor-Albert Chenevier, Plate-forme de Resources Biologiques, Créteil, France; 12 Institut National de la Santé et de la Recherche Médicale Centre d'Investigation Clinique 006, Hôpital Henri Mondor-Albert Chenevier, Pôle Recherche Clinique Santé Publique, Créteil, France; 13 Department of Biomedicine and the Centre for Integrative Sequencing, Aarhus University, Aarhus, Denmark; 14 Département de Psychiatrie et de Psychologie Clinique, Centre Hospitalier Universitaire de Nancy, Hôpital Jeanne-d'Arc, Toul, France; 15 Commissariat à l'Energie Atomique, Institut Génomique, Centre National de Génotypage, Evry, France; 16 Department of Psychiatry and Psychotherapy, University Medical Center, Georg-August-Universität, Göttingen, Germany; 17 Unit on the Genetic Basis of Mood and Anxiety Disorders, National Institute of Mental Health, National Institutes of Health, US Department of Health and Human Services, Bethesda, MD, United States of America; 18 Department of Psychiatry, University of California San Diego, La Jolla, CA, United States of America; 19 MRC Centre for Neuropsychiatric Genetics and Genomics, School of Medicine, Cardiff University, Heath Park, Cardiff, United Kingdom; 20 Assistance Publique - Hôpitaux de Paris, Groupe Hospitalier Lariboisière-F. Widal, Pôle de Psychiatrie, Paris, France; 21 Université Paris Diderot, Paris, France; University of Illinois at Chicago, United States of America

## Abstract

Bipolar disorder is one of the most common and devastating psychiatric disorders whose mechanisms remain largely unknown. Despite a strong genetic contribution demonstrated by twin and adoption studies, a polygenic background influences this multifactorial and heterogeneous psychiatric disorder. To identify susceptibility genes on a severe and more familial sub-form of the disease, we conducted a genome-wide association study focused on 211 patients of French origin with an early age at onset and 1,719 controls, and then replicated our data on a German sample of 159 patients with early-onset bipolar disorder and 998 controls. Replication study and subsequent meta-analysis revealed two genes encoding proteins involved in phosphoinositide signalling pathway (*PLEKHA5* and *PLCXD3*). We performed additional replication studies in two datasets from the WTCCC (764 patients and 2,938 controls) and the GAIN-TGen cohorts (1,524 patients and 1,436 controls) and found nominal *P*-values both in the *PLCXD3* and *PLEKHA5* loci with the WTCCC sample. In addition, we identified in the French cohort one affected individual with a deletion at the *PLCXD3* locus and another one carrying a missense variation in *PLCXD3* (p.R93H), both supporting a role of the phosphatidylinositol pathway in early-onset bipolar disorder vulnerability. Although the current nominally significant findings should be interpreted with caution and need replication in independent cohorts, this study supports the strategy to combine genetic approaches to determine the molecular mechanisms underlying bipolar disorder.

## Introduction

Numerous genome-wide association (GWA) studies have recently been performed on bipolar disorder (BD), but few signals were replicated and meta-analyses identified only a couple of associated genes, such as *CACNA1C*, *ODZ4* and *NCAN*, with a small effect size [1]–[4]. Although convergent evidences argue in favour of a role for these genes in vulnerability to BD [5]–[9], more extensive studies suggest it might be involved in other psychiatric disorders [3], [10], [11]. The difficulty to identify susceptibility genes specific to BD could be due to the ethnic heterogeneity, as a consequence of the huge number of patients and controls required to get genome-wide significant signals, or to the clinical and a genetic heterogeneity of the DSM-IV “bipolar disorder” entity.

In the current study, we focused on patients with BD selected with an early age at onset, a clinical characteristic known to identify a subgroup of patients with a higher morbid risk for BD in relatives [12]–[17]. We then compared these data to those observed in two previously published GWA studies of BD, conducted by the Wellcome Trust Case Control Consortium (WTCCC) [18] and the Genetic Association Information Network Bipolar Sample (GAIN-BP) [19]. Finally, we screened our regions of interest for rare variants, suspected to have a larger effect size, and suggested the importance of combined approaches in the identification of vulnerability genes to BD.

## Materials and Methods

### Ethics Statement

Protocols and procedures were approved by the research ethics board of the Pitié-Salpêtrière Hospital in Paris for the French sample and the ethics committees of the Faculties of Medicine at the Universities of Bonn and Mannheim/Heidelberg for the German sample. Written informed consent was obtained from all subjects prior to study participation.

### Sample

#### French sample

Two hundred and twenty patients of French descent with at least three grandparents from mainland France were collected through a French national network for mental health (Fondation FondaMental) in three university-affiliated psychiatry departments (Paris-Créteil, Bordeaux and Nancy) as previously described [12]. All patients met DSM-IV criteria [20] for BD type I (N = 172), type II (N = 46) or BD not otherwise specified (N = 2) before the age of 22. The age at onset was defined by the first mood episode (depressive, manic or hypomanic) and was retrospectively assessed using medical case notes and information obtained with the Diagnostic Interview for Genetic Studies (DIGS) [21]. The threshold of 22 was chosen on the basis of previous admixture analyses [16], [22]–[25].

#### German sample

One hundred and sixty-seven German patients were recruited from consecutive admissions to the psychiatric inpatient units of the Central Institute of Mental Health in Mannheim and the Department of Psychiatry and Psychotherapy of the University of Bonn (BOMA bipolar study). These patients received a lifetime diagnosis of BD type I based on DSM-IV criteria and had an age at onset lower than 22.

#### Control population

Genotyping data from 1,823 French controls and 629 German controls were provided by the *Centre National de Génotypage* (CNG, Evry, France). These samples were previously collected and were not screened for psychiatric disorders. They have been genotyped for association and population studies as described elsewhere [26] and permission to use them were obtained from the original investigators. DNA from 380 additional German controls were collected in the above mentioned German hospitals. An additional sample of 293 unaffected French controls screened for personal history of psychiatric disorders using the DIGS has been screened for mutation in the *PLCXD3* exon 2, in order to determine the specificity of the p.R93H mutation.

### DNA extraction and genotyping

DNA was isolated from lymphocytes either directly from venous blood sample or after transformation by Epstein Barr virus. Isolation was performed by salting-out with saturated sodium chloride solution [27]. French and German cases were genotyped at the CNG, using HumanHap550 or Human 610-Quad BeadArrays and the Infinium II assay (Illumina, San Diego, CA, USA). Control samples were genotyped on HumanHap300 (N = 2,503) or on HumanHap550 BeadArrays (N = 380). In order to determine whether the use of multiple arrays might result in a population stratification, we performed a multidimensional scaling analysis based on pairwise identity-by-state distance between pairs of individuals. The good clustering of all individuals showed that there was no stratification due to the use of multiple arrays (Figure S1 in File S1).

### Quality control criteria

All samples were passed through the standard quality-control procedures followed at the CNG for GWA studies, as described elsewhere [26]. For the French population, the quality control was performed using the PLINK toolset [28] on a total of 317,131 commons SNPs genotyped in 2,043 DNA samples (Table S1 in File S1). We removed monomorphic SNPs, as those with a minor allele frequency (MAF) lower than 0.01 and SNPs with a call rate lower than 0.97. We performed a missing chi-square test that compares, for each SNP, missingness between cases and controls and excluded SNPs with *P*<10^−3^. Then, we removed SNPs that did not show Hardy-Weinberg proportions in controls, using a significant threshold at *P*<10^−3^. In parallel, we removed male samples with more than 0.5% or female samples with less than 20% heterozygous markers on the X chromosome and individuals with a call rate lower than 0.97. We estimated the average genome-wide identity-by-state (IBS) sharing between individuals and analysed the clustering using a multidimensional scaling (MDS) plot. Hence, we excluded 15 individuals (3 cases and 12 controls) with a low level of identity. After pruning, the final data set consisted in 261,525 SNPs genotyped in 1,930 individuals (211 cases and 1,719 controls) that were used for association study. Identical quality control criteria were used for the replication sample from Germany (Table S1 in File S1) and MDS plot showed 6 controls to be removed. This led to a final data set of 288,167 SNPs and 1,157 individuals (159 patients and 998 controls).

### Association analysis

Basic case/control association analyses were performed using the PLINK software v1.07 [28]. A haplotype analysis was performed on the two most associated regions using sliding windows of two to five SNPs shifting by one SNP at a time. We tested for case/control haplotype-based association using a chi-square test with one degree of freedom. Only window with best *P*-values are reported for each region. Imputation data were based on the 3,967,651 SNPs genotyped in 90 subjects of the CEU population of the HapMap project (http://www.hapmap.org). Imputation was performed using PLINK and consisted in estimating the allele frequencies of an ungenotyped SNP based on its surrounding haplotypic background.

Genotyping data from the WTCCC [18] and the GAIN-TGen collection [19] were provided for BD patients with an age at onset lower than 22 and for controls. In the WTCCC, BD was defined according to Research Diagnostic Criteria [29] and included BD type I, type II, schizoaffective disorders and manic disorders. The WTCCC sample was genotyped on Affymetrix 500K array, containing only few SNPs in common with the Illumina arrays used in other samples. Thus, the genotype correlation data were obtained for SNPs within a 1 Mbp window, based on individuals and SNPs in the CEU HapMap filtered set (http://www.hapmap.org) and the 2,938 WTCCC control sample combined [18]. An association test between 764 early-onset patients and 2,938 controls was performed, using SNPs with a genotyped correlation higher than 0.2 with associated SNPs at *P*<5×10^−5^ identified through the meta-analysis.

All patients from the GAIN-TGen cohort met DSM-IV criteria for BD type I. Imputed data from the GAIN-TGen bipolar case-control sample [30] were obtained, using the MACH software with phased haplotypes from the 60 HapMap-CEU founders (release 22). Allele frequencies of 15 SNPs out of the 16 associated SNPs at *P*<5×10^−5^, were compared between 1,524 patients with an early age at onset and 1,436 controls.

A flow chart summarizing all steps of the genetic analysis is shown on the Figure S2 in File S1.

### Detection of copy number variations

Copy number variations were detected by fluorescence level comparison through the chromosome, using the SnipPeep software (R. Toro, Pasteur Institute, France). The deletion in *PLCXD3* was confirmed by quantitative PCR analysis using one internal and two external couples of primers and Mesa Green qPCR master mix plus (Eurogentec, Liege, Belgium). PCR were performed and run in a Mastercycler ep realplex2S (Eppendorf, Hamburg, Germany) for the deleted patient and two unaffected controls, known to have no deletion in the regions under study. Each real-time PCR was performed in triplicate and assessed by comparing Ct at a determined threshold between the three amplicons and the three individuals.

### Mutation screening in PLCXD3

The *PLCXD3* coding regions were amplified for 334 French subjects with BD, sequenced by the Sanger's method using the BigDye terminator v3.1 cycle sequencing kit (Life Technologies, Carlsbad, CA, U.S.A.) and run on a 16-Capillary ABI PRISM 3130xl genetic analyzer. The *PLCXD3* exon 2 was sequenced for 293 unaffected French controls. All primers used for PCR amplifications and sequence analyses are available on request.

## Results

### Analysis of a French cohort

We carried out an association study on 211 patients with early-onset BD of French origin and 1,719 matched controls, who have been genotyped on an Illumina platform with 261,525 single nucleotide polymorphisms (SNPs) passing quality control. The estimation of an inflation factor [31] showed a good genetic homogeneity in our sample (λ = 1.03), suggesting that no correction for genomic control was needed. According to the genome-wide significance threshold of *P* = 5×10^−8^, no significant difference in allele frequencies was observed in our sample, but 17 SNPs showed a nominal *P*<5×10^−5^ (Table S2 in File S1), suggesting these loci may be associated with vulnerability to early-onset forms of BD. Interestingly, the lowest *P*-value (*rs10096683*, *P* = 4.11×10^−6^, OR = 2.26), was observed for a SNP located on chromosome 8p22, a region that has previously been associated with schizophrenia [32]–[35] and suicide behaviour in patients with recurrent major depression [36].

### Replication on a German cohort

Next, we selected 14,037 SNPs with a *P*<0.05 to perform a replication study on an independent sample of 159 German subjects with an early-onset BD and 998 matched controls (Table S1 in File S1). Again, a good genetic homogeneity (λ = 1.04) was observed after quality control for this sample. Out of the 14,037 SNPs previously selected, 13,734 were genotyped in the German sample, and 739 showed a *P*<0.05, showing a significant over-representation (*P*<0.03) of nominally significant *P*-values. The most associated SNP in the German sample was located on chromosome 12 upstream to the *PLEKHA5* gene (*rs2970836*, *P* = 5.3×10^−6^, OR = 1.76).

### Meta-analysis on the French and the German cohorts

**Figure 1:**
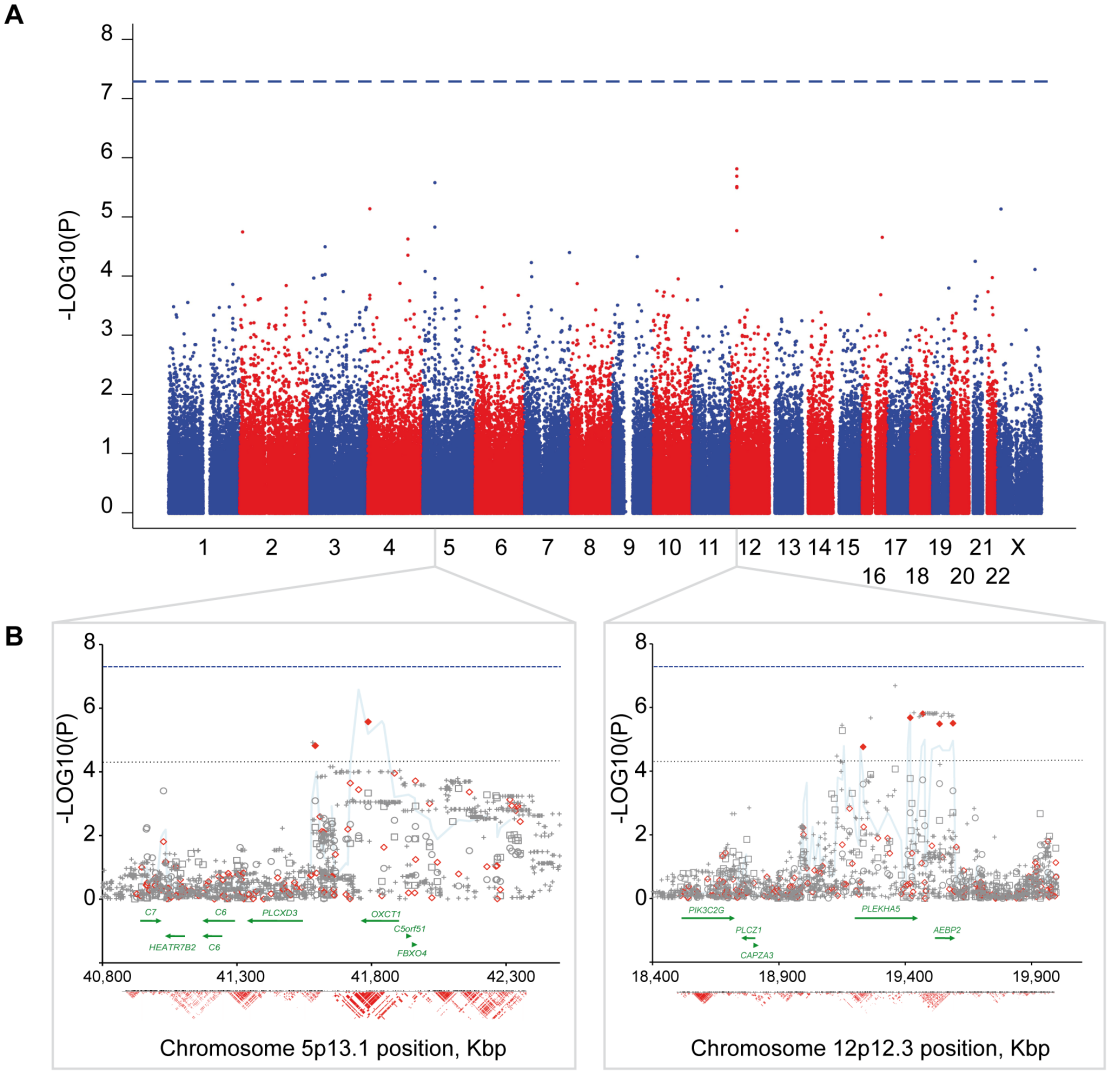
Genome-wide association results and detailed peak association regions. (**A**) Manhattan plot of the meta-analysis performed on early-onset bipolar patients and controls from France and Germany. Physical position is shown along the *x* axis and –log10(*P*-value) is shown along the *y* axis. (**B**) Detail of the two most associated regions on chromosomes 5p13 and 12p12. Allele frequency differences are represented by –log10(*P*-values) for the French (open grey circles), the German (open grey squares) and the meta- (open red diamonds) analyses. Grey crosses represent –log10(*P*-value) for imputed ungenotyped SNPs. The most associated SNP for each region is shown with orange circle. On chromosome 12p12, the lowest *P*-value (*P* = 2.1×10^−7^) was observed for an imputed SNP (*rs10743315*). On chromosome 5p13, the lowest *P*-value (*P* = 2.6×10^−7^) was observed for a three-SNPs window haplotype (light blue line) located downstream to *OXCT1* and upstream to *PLCXD3* (*rs624097-rs316762-rs10512793*). The genome-wide significant threshold (*P* = 5×10^−8^) is indicated by the blue dash line and the dot black line shows a threshold at *P* = 5×10^−5^. The largest differences in allele frequencies are represented with filled diamonds. Gene position and annotation (http://genome.ucsc.edu/) are symbolised by green arrows. Linkage disequilibrium (r^2^) estimated according to HapMap CEU population SNPs (release 3) is symbolised in the bottom part of each figure. Darker red indicates higher values.

We subsequently performed a meta-analysis, combining data from the French and the German cohorts, yielding a sample of 370 patients with early-onset BD and 2,717 controls. Again, no locus reaches the genome-wide significant threshold. The largest difference in allele frequencies between cases and controls was observed for chromosome 12 (*rs2961365*, *P*_meta_ = 1.6×10^−6^, OR_meta_ = 1.57) with no evidence of heterogeneity between the two cohorts (Q = 0.94, 1 degree of freedom, I^2^ = 0). In this region, 5 SNPs showed a difference in allele frequencies at *P*<5×10^−5^ in 355 Kbp (Figure 1, Table 1). These SNPs span two genes, *PLEKHA5* and *AEBP2*. The second strongest association with no heterogeneity (Q = 0.59, 1 degree of freedom, I^2^ = 0) was observed for chromosome 5p13 (*rs10512793*, *P*_meta_ = 2.7×10^−6^, OR_meta_ = 1.61), in which two SNPs, spaced by 196 Kbp, showed a difference in allele frequencies at *P*<5×10^−5^ (Figure 1, Table 1). These two SNPs were located in the *OXCT1* gene, and upstream to *PLCXD3*. In order to further explore the associated peak in these two regions, we used a three-SNP sliding window and performed a haplotype analysis. Whereas no further significant difference, as compared to single SNP analysis, was observed on chromosome 12p12, a more significant difference between patients and controls was observed on chromosome 5p13 for the combination of *rs624097-rs316762-rs10512793*, for which an overall *P*_meta_ = 2.6×10^−7^ was observed (Figure 1).

**Table 1:** Regions associated at *P*<5×10^−5^ with early-onset BD in meta-analysis.

				French sample				German sample				Meta-analysis			
CHR	SNP	Position^a^ (bp)	Allele	Allele frequency in early-onset BD patients	Allele frequency in controls	*P*	OR	Allele frequency in early-onset BD patients	Allele frequency in controls	*P*	OR	*P*_meta_	OR_meta_	Q	I^2^
12	*rs2961365*	19,469,374	T	0,23	0,16	1,88×10^−04^	1,58	0.23	0.16	2.12×10^−03^	1.56	1.58×10^−6^	1.57	0.94	0
12	*rs3752823*	19,419,905	A	0,15	0,10	2,03×10^−03^	1,58	0.16	0.09	1.70×10^−04^	1.89	2.09×10^−6^	1.71	0.43	0
5	*rs10512793*	41,787,459	C	0,19	0,13	1,24×10^−03^	1,54	0.20	0.13	4.64×10^−04^	1.72	2.68×10^−6^	1.61	0.59	0
12	*rs2900433*	19,588,898	T	0,23	0,16	4,13×10^−04^	1,55	0.23	0.16	2.01×10^−03^	1.57	3.13×10^−6^	1.56	0.96	0
12	*rs7305762*	19,535,793	G	0,23	0,16	2,46×10^−04^	1,57	0.23	0.16	3.53×10^−03^	1.53	3.26×10^−6^	1.55	0.89	0
4	*rs12500759*	7,760,780	T	0,21	0,29	7,54×10^−04^	0,66	0.23	0.32	2.76×10^−03^	0.66	7.40×10^−6^	0.66	1.00	0
X	*rs952076*	13,856,877	T	0,20	0,28	2,61×10^−03^	0,65	0.19	0.29	5.94×10^−04^	0.56	7.48×10^−6^	0.61	0.51	0
5	*rs822135*	41,590,915	A	0,25	0,33	8,12×10^−04^	0,68	0.26	0.34	5.54×10^−03^	0.69	1.50×10^−5^	0.68	0.94	0
12	*rs4764470*	19,233,420	A	0,27	0,20	2,55×10^−04^	1,53	0.25	0.20	1.84×10^−02^	1.39	1.73×10^−5^	1.47	0.60	0
2	*rs7608161*	9,668,060	G	0,34	0,27	2,90×10^−03^	1,38	0.33	0.25	1.68×10^−03^	1.50	1.82×10^−5^	1.43	0.64	0
16	*rs7185187*	69,315,056	T	0,11	0,06	5,99×10^−04^	1,80	0.12	0.07	9.63×10^−03^	1.65	2.24×10^−5^	1.73	0.74	0
4	*rs6857347*	139,528,195	G	0,43	0,50	6,80×10^−03^	0,75	0.41	0.51	7.62×10^−04^	0.66	2.40×10^−5^	0.71	0.41	0
3	*rs13091545*	52,352,334	G	0,25	0,18	1,73×10^−04^	1,57	0.20	0.16	4.64×10^−02^	1.35	3.24×10^−5^	1.48	0.44	0
7	*rs4716990*	155,616,697	G	0,37	0,29	6,12×10^−04^	1,45	0.36	0.29	1.99×10^−02^	1.34	4.04×10^−5^	1.40	0.66	0
4	*rs12505439*	139,521,194	A	0,35	0,29	9,91×10^−03^	1,32	0.40	0.30	1.05×10^−03^	1.50	4.48×10^−5^	1.40	0.44	0
9	*rs3739657*	85,177,755	C	0,28	0,36	2,21×10^−03^	0,71	0.31	0.39	6.87×10^−03^	0.70	4.74×10^−5^	0.71	0.98	0

OR, odds ratio; Q, *P*-value for Cochrane's Q statistic; I^2^, heterogeneity index.

a position from the short arm telomere based on Hg18.

### Imputation data

In order to refine the association signal in these two regions, we imputed data for ungenotyped SNPs in the French and the German cohorts based on the 3.9 million SNPs genotyped in the CEU population of the HapMap project. The results of the two populations were then combined to perform an association study. After imputation, we observed an increased signal for chromosome 12 with the lowest estimated *P*-value for *rs10743315* (*P* = 2.1×10^−7^, OR = 1.61) located in *PLEKHA5* (Figure 1). On the contrary for chromosome 5, the lowest *P*-value was observed for the genotyped SNP, *rs10512793*.

### Replication studies on the WTCCC and the GAIN-TGen cohorts

We analysed the top-16 SNPs (*P*<5×10^−5^) out of the meta-analysis in two additional samples of early-onset bipolar patients, already genotyped on different platforms through the WTCCC and the GAIN and performed a mega-analysis including the four samples (Table S3 in File S2). Only 4 SNPs, genotyped in a subsample of 764 early-onset bipolar patients and 2,938 matched controls from the United Kingdom out of the WTCCC, were either directly or in strong linkage disequilibrium (r^2^>0.99) with SNPs genotyped in the WTCCC sample. None of them showed a nominal *P*-value (*P*<0.05). However, two SNPs on chromosome 5p13 in lower linkage disequilibrium (r^2^ = 0.65) with *rs822135* showed a nominal *P*-value (*rs625487*, *P* = 0.02, OR = 1.15 and *rs633407*, *P* = 0.02, OR = 1.15) (data not shown). Both were located approximately 55 Kbp upstream to *PLCXD3* and 165 Kbp downstream to *OXCT1*. In addition, one SNP (*rs2565666*), located on chromosome 12p12, 21 Kbp downstream to *PLEKHA5* and 42 Kbp upstream to *AEBP2*, also showed a nominal *P*-value (*rs2565666*, *P* = 0.03, OR = 1.22) (data not shown). We also compared a sample of 1,524 early-onset bipolar patients with 1,436 controls out of the combined GAIN-TGen cohorts. None out of the 15 tested SNPs showed a nominal *P*-value (Table S3 in File S2). The mega-analysis showed a high heterogeneity between the samples from France and Germany and the two samples from U.K. and U.S.A. This analysis resulted in a low signal on chromosomes 5p13 and 12p12 (*rs10512793*, *P_mega_* = 5.05×10^−2^, OR_mega_ = 1.38 and *rs3752823*, *P_mega_* = 5.79×10^−2^, OR_mega_ = 1.43, respectively). The strongest association observed in the mega-analysis for SNPs tested in at least three of the four populations was observed on chromosome 7q36.3 in a gene desert (*rs4716990*, *P_mega_* = 3.78×10^−2^, OR_mega_ = 1.25).

### Structural abnormalities in region of interest

**Figure 2:**
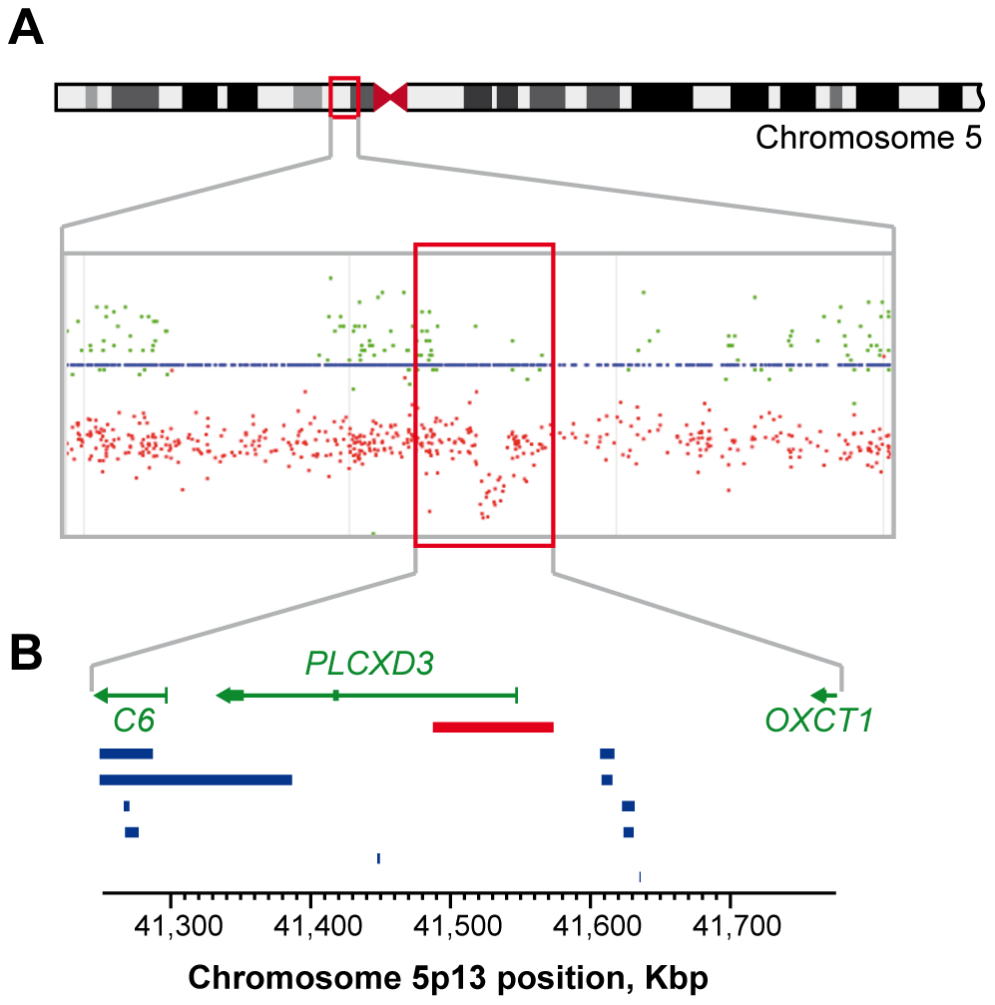
Copy number variation identified on chromosome 5p13. (**A**) Fluorescence level of SNPs of the region for the deleted patient visualized using SnipPeep. Red dots represent fluorescence level of SNPs and green dots correspond to the SNP heterozygocity. (**B**) Chromosomal position of the deletion. Gene position and annotation (http://genome.ucsc.edu/) are symbolised by green arrows. Deletions reported in the Database of Genomic Variants (http://projects.tcag.ca/variation/) are represented with blue bars. No duplication has been reported in this region. A red bar represents the deletion identified in the patient with early-onset BD.

Fluorescence level of genotyped SNPs was only available for a subsample of early-onset patients of French and German origin (N = 321). We analysed this fluorescence level in the two 1.5 Mbp regions of interest on chromosome 5p13 and 12p12. On chromosome 5p13, we identified, in a French patient who manifested a first mood episode by the age of 17, a 90-Kbp deletion removing the *PLCXD3* promoter region as well as the first coding exon of the gene (Figure 2), whereas no copy number variation has been previously reported in this region in the database of genomic variations (http://projects.tcag.ca/variation/). Although DNA was not available for first-degree relatives of this patient, the deletion was confirmed using quantitative PCR analysis (data not shown). In order to determine whether independent family specific mutations might be frequently found in bipolar patients, we screened all coding exons of *PLCXD3* in 334 French subjects (204 patients with early-onset BD and 130 patients with late-onset BD). Three synonymous variations (p.V58, p.A130 and p.P251) were found in these patients, and one amino acid change (p.R93H) was identified in one subject with early-onset BD type I and obsessive compulsive disorder. This variation was not found in 293 unaffected matched controls. This amino acid is highly conserved through evolution and, according to PolyPhen-2 [37], this variation is predicted to strongly affect the function of the protein. Further analysis of the available DNA sample in this family showed that this variation was transmitted from the father (Figure S3 in File S1). The patient manifested first mood symptoms by the age of 19. Although his father did not meet criteria for mood disorder according to DSM-IV, his first cousin manifested major depressive disorder by the age of 65. Unfortunately, no DNA was available for this subject.

## Discussion

The important clinical heterogeneity of BD has probably hampered the identification of vulnerability genes so far. After tumultuous starts, GWA studies have enabled the identification of robust and replicable genetic findings of weak effect, in regions of *CACNA1C*, *ODZ4* and *NCAN*, but without disease specificity [4]. In this study, we conducted a genome-wide approach on a highly heritable phenotype, focusing on patients with an early age at onset. Based on the genotyping data of 261,525 SNPs, we did not identify SNPs that reached the genome-wide significant threshold in our patients and controls of French origin. This was most likely due to the small sample size of our cohort. We calculated the statistical power of our study, using the CaTS software [38] and estimated that we had 80% chance to detect only SNPs with a genetic risk factor higher than 1.7 (Figure S4 in File S1). However, recent data from larger cohort studies showed a polygenic component in BD [3], [39], consistent with a combine vulnerability resulting from many variants of weak effect. We thus conducted a replication study on a German cohort of patients with early-onset BD and performed a subsequent meta-analysis. Although it did not reach the genome-wide significant threshold, the most associated region in our meta-analysis was located on chromosome 12p12. This region has been previously associated with bipolar disorder [40]–[42] and more specifically when patients had an early age at onset of mania [40]. Five SNPs in this region showed a difference in allele frequencies between patients and controls, spanning two genes, *PLEKHA5* and *AEBP2*. The genetic refinement using imputed data showed the lowest *P*-value for a SNP located in *PLEKHA5* (*rs10743315*, *P* = 2.1×10^−7^). *PLEKHA5* encodes a protein containing a pleckstrin homology domain, which interacts with phosphatidylinositol 3-phosphate [43]. Few elements are known about this gene except its ubiquitous pattern of expression, including foetal and adult brains. Nevertheless, there is compelling evidence for the implication of the phosphatidylinositol signalling pathway in the etiopathogeny of BD and the two most effective mood stabilisers for BD (lithium and valproate) directly inhibit this pathway [44]. Further exploration of response to mood stabilizer in regards to the *PLEKHA5* genotypes might help in understanding the difference observed according to the patients' age at onset [15].

The second most associated region in our meta-analysis was located on chromosome 5p13. In this region, the highest difference in allele frequencies between patients with early-onset BD and controls was observed for *rs10512793*. This SNP was located in *OXCT1*, which encodes a 3-oxoacid CoA transferase 1. This enzyme catalyzes the reversible transfer of CoA from succinyl-CoA to acetoacetate and is thus the first step of ketone body utilization, the main source of lipid-derived energy for the brain [45]. Mutations in this gene are associated with succinyl CoA:3-oxoacid CoA transferase (SCOT) deficiency, characterised by episodes of severe ketoacidosis [46], [47], which can also emerge during treatment with some atypical antipsychotics [48]. However, the haplotype analysis of this region suggested that the associated peak might be located more likely downstream to *OXCT1* and upstream to *PLCXD3*. This result is consistent with the 90 Kbp-deletion reported in one of the French patients and that removed the promoter region and the first coding exon of *PLCXD3*. Further exploration of this gene revealed a mutation predicting to change the amino acid sequence of the protein (p.R93H) and transmitted from his father, who had a first cousin with major depressive disorder. Interestingly, this gene encodes a phosphatidylinositol-specific phospholipase C, strengthening the involvement of the phosphoinositide-signalling pathway in vulnerability to BD. Note that tricyclic antidepressants (e.g. desipramine), which are known to induce rapid cycling or induce manic or hypomanic episodes in some patients with BD [49], stimulate phospholipase C activity and the production of the second messenger inositol 1,4,5-trisphosphate [50]. In addition, experiments performed using rat cultured hippocampal neurons revealed that desipramine rapidly enhanced the spontaneous SNAP25-dependent vesicular release of glutamate [51]. This Ca^2+^ dependent mechanism is consistent with our previously reported association between *SNAP25* and early-onset BD [12] and with the strong associations observed between SNPs located in *CACNA1C* and BD [1], [3].

The main limitation of our study is not to reach the stringent genome-wide threshold. Although this might translate an absence of common genetic variations for a clinical subgroup based on an early age at onset, this is also a direct consequence of the small sample size and the loss of power inherent to the subphenotype analysis. Similar study on larger sample should be performed to confirm our data and valid the relevance of such approach. Nevertheless, results observed in our French subsample with an early age at onset have been replicated in a German sample and supported by the data from the WTCCC with similar phenotype criteria.

Another possible cause of the loss of power in our analysis is the categorical exploration of the age at onset. Despite a widely valid threshold for the age at onset [16], [17], [22]–[25], a continuous exploration of this variable would allow to determine whether specific loci might lead to various age at onset, which would result, under multiplicative or additive models, in an overall early age-at-onset in patients. Such hypothesis is supported by the recent observation of the International Schizophrenia Consortium, which showed the molecular genetic evidence for a substantial polygenic component to the risk of BD involving thousands of common alleles of very small effect [39]. Similar analysis should thus be performed according to the patients' age at onset, but this should require larger samples for the study and the replication analysis.

In conclusion, although we did not reach the genome-wide significant threshold in our association study, we report here convergent data between our GWA study on early onset BD, the structural abnormality observed in one patient and a missense mutation reported in one family with early-onset BD. The difficulties to reach the stringent genome-wide significant threshold suggest that larger samples should be included, which will also help in better defining the most convenient strategy to take into account the age at onset of patients. Although the current nominally significant findings should be interpreted with caution and need replication in independent cohorts, our results displayed that the combined exploration of common and rare variants in BD can reveal identical pathways suggesting both approaches should be systematically used in further genetic exploration of this disorder. Using these approaches, we provided evidence that variations in phosphoinositide secondary messenger signalling pathway, and for the first time, that the *PLEKHA5* and *PLCXD3* genes, might confer vulnerability to early-onset BD. Further exploration of these mechanisms might be used to better identify markers of prognosis and help the development of innovative treatment for these severe forms of the disease.

## Supporting Information

File S1 **Contains Tables S1-S2 and Figures S1-S4.**
(PDF)

File S2 **Contains Table S3.**
(XLSX)
