# Supplementary material for: Common and Rare Variant Analysis in Early-Onset Bipolar Disorder Vulnerability
Source: PLoS One. 2014 Aug 11;9(8):e104326. doi: 10.1371/journal.pone.0104326 (PMC4128749; doi:10.1371/journal.pone.0104326)
Supplement: File S1 — Contains Tables S1-S2 and Figures S1-S4. (PDF) [file pone.0104326.s001.pdf]

Supporting Information

Table S1. Quality control criteria used for the French and the German populations

	French			German		
	Patients		Controls	Patients	Controls	
	HH550	H610Q	HH300	HH550	HH550	HH300
Number of SNPs at start of QC	561,466	582,892	318,237	561,466	561,466	318,237
Common SNPs between HumanHap300, HumanHap550 and Human610-Quad BeadArrays	317,131	317,131	317,131	312,772	312,772	312,772
SNPs wit call rate < 0.97	20,467		35,696	15,301	2,622	10,848
SNPs with MAF < 0.01	236		242	300	263	245
SNPs with HWE failure *P* < 10^{-3}	377		870	295	600	
SNPs with PLINK chi-missing test P < 10^{-3}	7,649			2,157		
**Number of SNPs at end of QC**	**261,525**			**288,167**		
Number of individuals at start of QC	165	55	1,823	167	380	629
Individuals with < 0.97 call rate	4		87	3	5	
Sex discordance	2		5	5	0	
Population stratification gross outliers	3		12	0	6	
**Number of individuals at end of QC**	**211**		**1,719**	**159**	**998**	

HH300, HumanHap300; HH550, HumanHap550; H610Q, Human-610-Quad

Table S2. Region associated at $P < 5 \times 10^{-5}$ with early-onset BD in the French population

CHR	SNP	*P*-value	Position (bp)	type	Reference allele	Closest genes in 200 kbp (upstream; downstream)
8	rs10096683	4.11×10^{-6}	14,206,574	intronic	G	*SGCZ*
1	rs12031354	4.60×10^{-6}	161,128,971	intergenic	G	*C1orf110; RGS4*
4	rs763532	8.55×10^{-6}	36,913,244	intergenic	T	*-; KIAA1239*
1	rs1108610	9.99×10^{-6}	20,601,633	within non coding gene	G	*BC040588*
8	rs1504772	1.78×10^{-5}	4,087,225	intronic	A	*CSMD1*
X	rs5930575	1.83×10^{-5}	131,637,050	intronic	A	*HS6ST2*
20	rs6028791	1.94×10^{-5}	38,078,497	intergenic	C	*BQ417071; BQ574468*
21	rs2829656	1.94×10^{-5}	25,538,071	within spliced EST	T	*CD365222*
14	rs8019549	1.95×10^{-5}	36,766,730	5’-UTR	A	*MIPOL1*
5	rs34116	2.02×10^{-5}	80,477,755	intronic	T	*RASGRF2*
3	rs291504	2.11×10^{-5}	73,956,783	within spliced EST	G	*AW295978, BU188154*
14	rs10135285	2.13×10^{-5}	36,757,591	intronic	G	*MIPOL1*
2	rs1250062	2.14×10^{-5}	216,250,506	within non coding gene	T	*AK124897; AK091865*
21	rs2829644	2.34×10^{-5}	25,508,195	within spliced EST	A	*CD365222*
15	rs1393941	2.50×10^{-5}	98,881,024	intronic	C	*LASS3*
4	rs12645446	4.10×10^{-5}	105,183,194	intergenic	C	*-; -*
6	rs823692	4.50×10^{-5}	7,435,616	intergenic	C	*RIOK1; DSP*

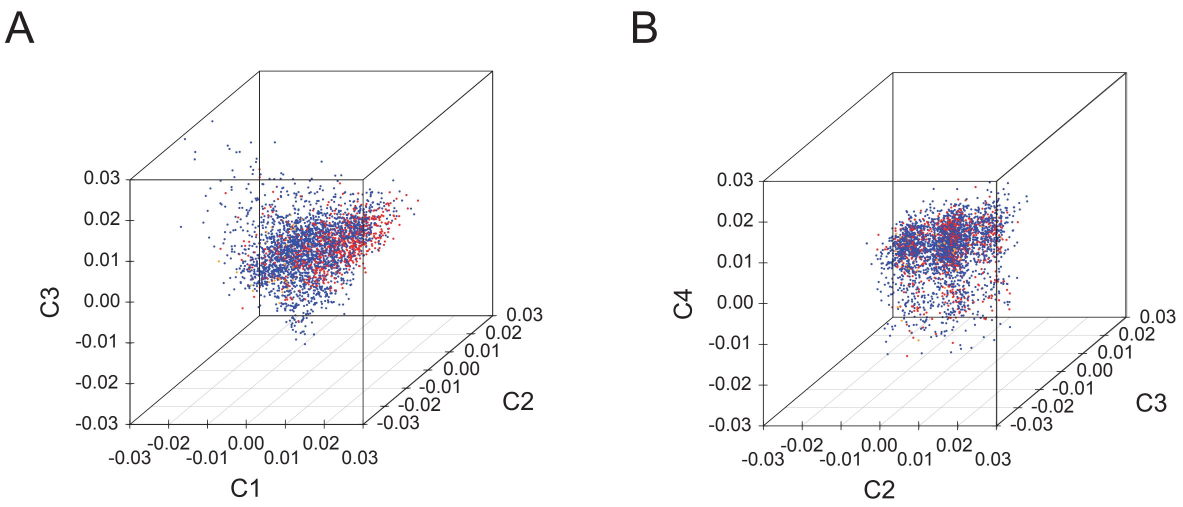

Figure S1. Multidimensional scaling (MDS) plot for the French and German patients and controls. The first four dimensions of a MDS analysis based on pairwise identity-by-state distance between pairs of individuals are represented in **A** (C1, C2 and C3) and **B** (C2, C3 and C4). Each dot represents an individual with blue dots corresponding to subjects genotyped with Illumina HumanHap300 BeadChips, red dots corresponding to subjects genotyped with Illumina HumanHap550 BeadChips and orange dots corresponding to subjects genotyped with Illumina Human610-Quad BeadChips. No difference between arrays was observed on these four dimensions.

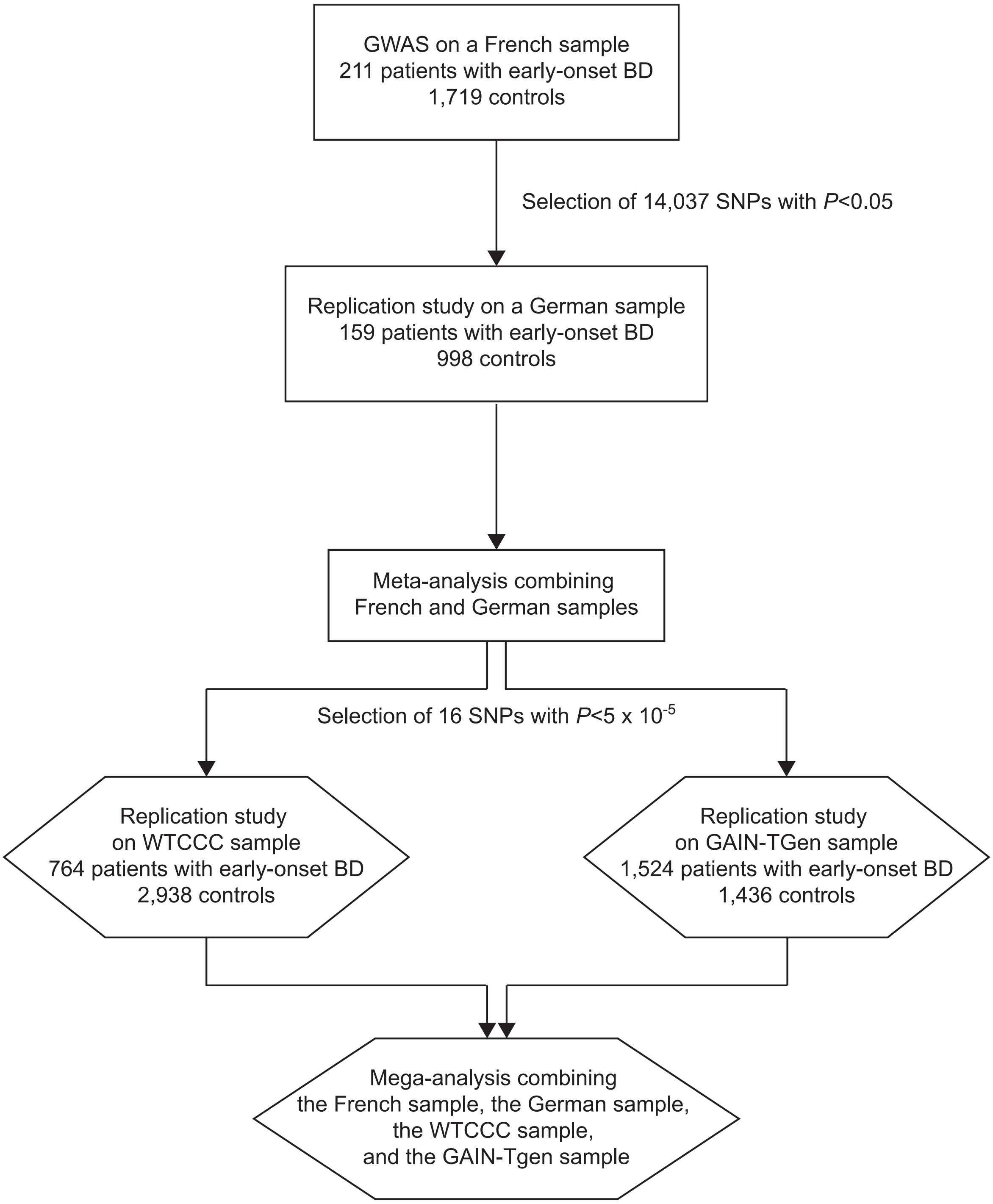

Figure S2. Flow chart summarizing the procedural analysis process on the different cohorts used in this study.

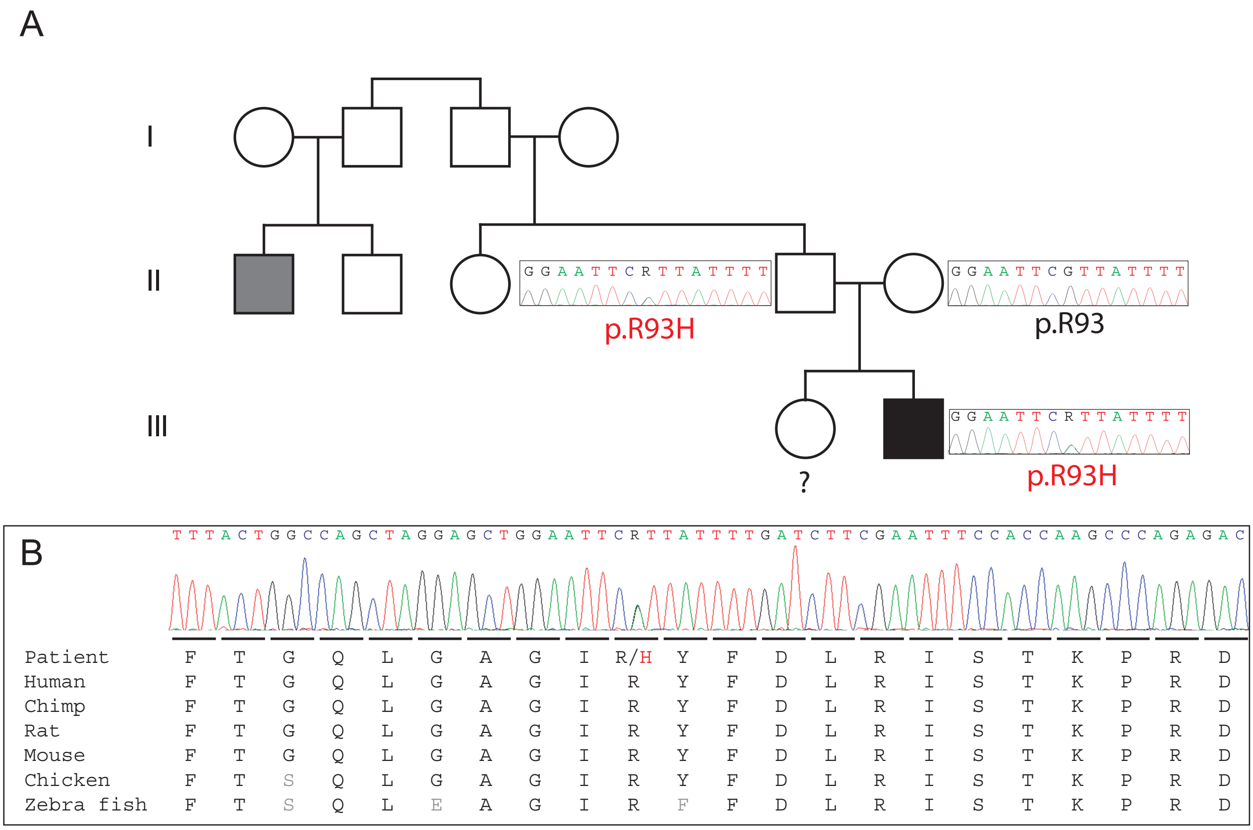

Figure S3. Identification of the p.R93H mutation in *PLCXD3*. A. Family pedigree of the patient carrying the *PLCXD3* p.R93H mutation. Males are indicated with squares, females are indicated with circles. Affected subjects with bipolar disorder are indicated in black, and those with major depressive disorder are indicated in grey. **B.** Chromatogram corresponding to the DNA sequence observed in patient as well as the PLCXD3 protein alignment in six vertebrates. The amino acid affected by the p.R93H mutation is highly conserved through evolution.

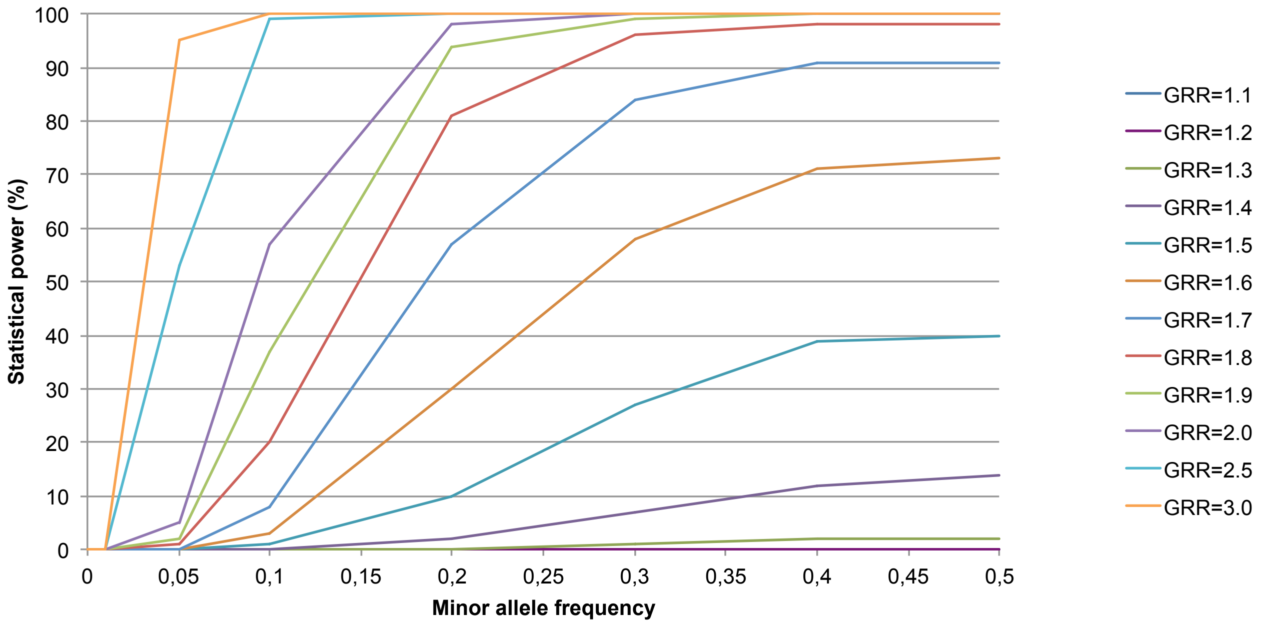

Figure S4. Estimation of the statistical power. The statistical power calculation was based on 211 cases and 1,719 controls for the discovery sample and 159 cases and 998 controls for the replication sample. 5% of SNPs were genotyped for the replication sample. We considered a BD prevalence of 0.01 and a genome-wide significance threshold of $P = 5 \times 10^{-8}$. GRR, genotype relative risk.
